# Supplementary material for: The interactome of intact mitochondria by cross-linking mass spectrometry provides evidence for coexisting respiratory supercomplexes
Source: Mol Cell Proteomics. 2017 Dec 8;17(2):216–32. doi: 10.1074/mcp.RA117.000470 (PMC5795388; doi:10.1074/mcp.RA117.000470)
Supplement: Supplemental Data [file supp_RA117.000470_133922_0_supp_23978_fzffwf.zip › spectra_annotation/mito_DR_spectra_annotation/410-1-3-1-2-1.pdf]

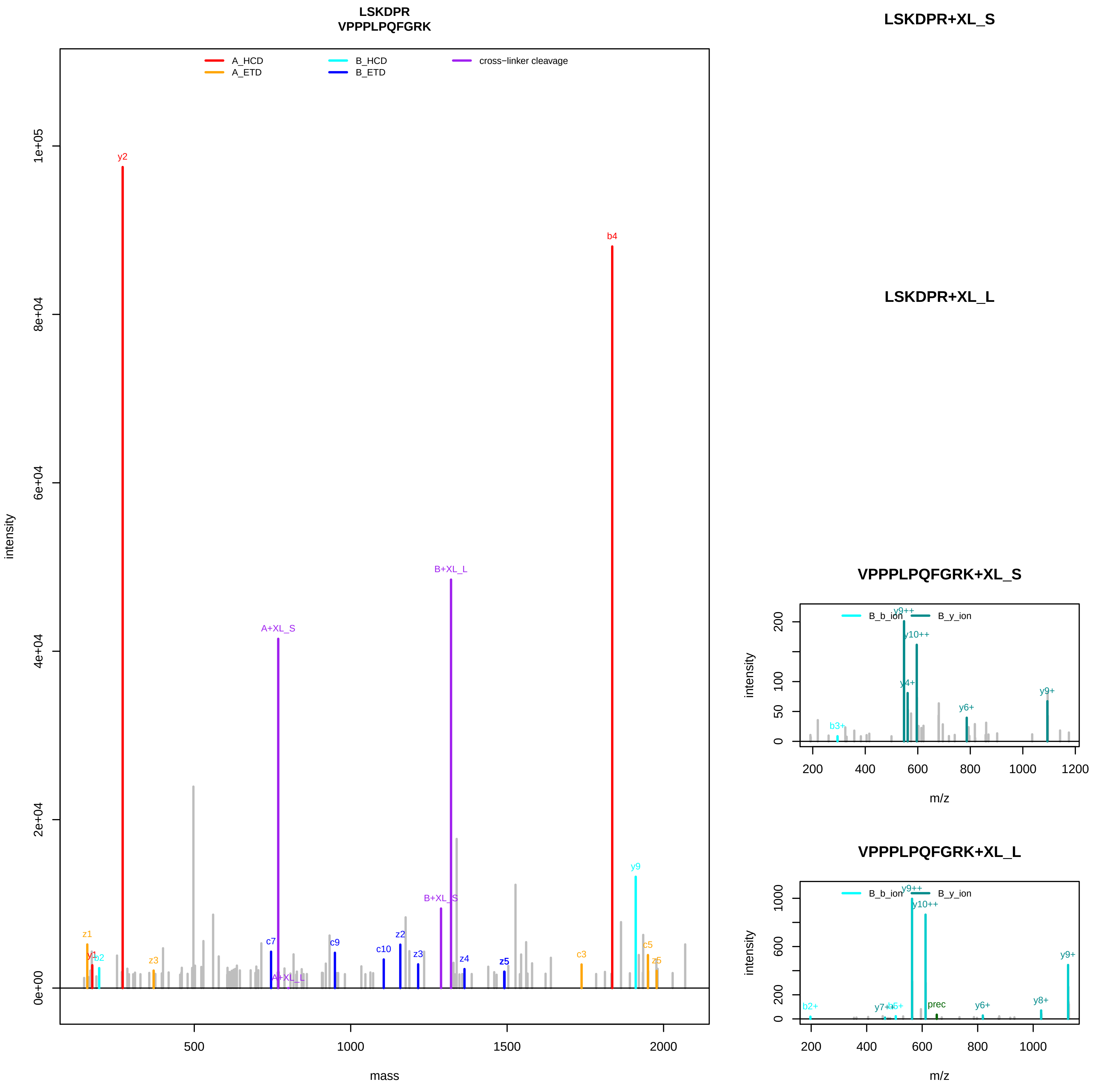

LSKDPR
VPPPLPQFGRK
A_HCD
A_ETD
B_HCD
B_ETD
cross−linker cleavage
intensity
mass
0e+00
2e+04
4e+04
6e+04
8e+04
1e+05
500
1000
1500
2000
y2
b4
B+XL_L
A+XL_S
B+XL_S
A+XL_L
y9
z1
y1
b2
z3
c7
c9
c10
z2
z3
z4
z5
c3
c5
z5
LSKDPR+XL_S
LSKDPR+XL_L
VPPPLPQFGRK+XL_S
B_b_ion
B_y_ion
y9++
y10++
y4+
y6+
y9+
b3+
m/z
VPPPLPQFGRK+XL_L
y8+
y7++
prec
b2+
